# Assessing the Environmental and Occupational Health Implications of Styrene Emissions in Cured-In-Place Pipe (CIPP) Rehabilitation: A Multi-Site Analysis of Installation Practices

**DOI:** 10.3390/ijerph22101543

**Published:** 2025-10-09

**Authors:** Parisa Beigvand, Mohammad Najafi, Vinayak Kaushal, Ayoub Mohammadi, William Elledge, Burak Kaynak

**Affiliations:** 1 Center for Underground Infrastructure Research and Education (CUIRE), Department of Civil Engineering, University of Texas at Arlington, P.O. Box 19308, Arlington, TX 76019, USA; najafi@uta.edu (M.N.); vinayak.kaushal@uta.edu (V.K.); axm5872@mavs.uta.edu (A.M.); 2 DC Water and Sewer Authority, Washington, DC 20003, USA; william.elledge@dcwater.com (W.E.); burak.kaynak@dcwater.com (B.K.)

**Keywords:** environmental health impact, styrene emissions, occupational exposure, trenchless technology, pipeline rehabilitation

## Abstract

Styrene is an aromatic compound widely used as a reactive monomer in polyester resins, which are among the most utilized resins in cured-in-place pipe (CIPP) technology, the most widely used trenchless pipe renewal method. Given that styrene is classified as a suspected human carcinogen, this study aims to evaluate styrene concentrations emitted into the air during sewer pipe rehabilitation using CIPP. This study included developing a comprehensive methodology to collect data from six different CIPP installations across the U.S. and document styrene emissions before, during, and after the curing process. The air samples were collected and analyzed using the USEPA method TO-15 and TO-17. Measured styrene emissions were then compared with exposure limits established by USEPA, NIOSH, and OSHA to assess potential occupational and worker health impacts. The result confirmed that high styrene concentrations, exceeding the established threshold, can be observed within the CIPP work zone. The result also indicated a considerable reduction in styrene concentration within five feet downwind of the work zone. In conclusion, while the health risk to the public appears to be low, there is a potential for health impact for the CIPP crew. Therefore, implementing real-time air quality monitoring during CIPP installation to mitigate these health risks is recommended. Additionally, the use of appropriate personal protective equipment (PPE) by the crew is essential.

## 1. Introduction and Background

Cured-in-place pipe (CIPP) installation is one of the most widely used trenchless methods for pipeline rehabilitation, having been introduced in the 1970s. It is commonly used to repair deteriorated sanitary sewers, stormwater, and drinking water pipelines. The CIPP process involves inserting a liquid thermoset resin-saturated tube, composed of either styrene or non-styrene-based resins, into a damaged pipeline. The liner conforms to the shape of the host pipe and adheres to its inner surface using hydrostatic pressure, air inversion, or mechanical pulling and inflation, and then is cured in place utilizing hot water, steam, or ultraviolet (UV) light [1].

The resins utilized in CIPP installations include polyester, vinyl ester, and epoxy. Styrene monomer serves dual functions in the formulation of vinyl ester resin (VER) composites, acting both as a reactive diluent to reduce resin viscosity and as a cross-linking agent that enhances mechanical strength [2]. Styrene-based resins such as polyester and vinyl ester are the most widely used due to their cost-effectiveness and ease of application [3]. CIPP liners for gravity pipelines are typically constructed from polyester needle-punched felt or reinforced tubes saturated with isophthalic polyester or vinyl ester thermoset resins containing styrene. Styrene content in neat and filled polyester resins can reach 40% and 30% by weight, respectively [4].

Styrene, a commonly used volatile organic compound (VOC) in polyester resins, enhances the durability and strength of the liners. Exposure to styrene poses both respiratory and neurological health risks. In addition, styrene emissions contribute to environmental pollution, raising concerns about air quality in areas surrounding workplaces [5]. Styrene, classified as a Hazardous Air Pollutant (HAP) by the U.S. Environmental Protection Agency [6], is reasonably anticipated to be a human carcinogen. Most evidence of styrene carcinogenicity in humans comes from occupational studies in the reinforced plastics and styrene–butadiene rubber industries [7].

Matthews et al. [8] identified styrene as the primary chemical of concern in CIPP installations, noting that measured concentrations—derived from both field data and modeling analyses—have the potential to pose health risks. However, due to methodological limitations in existing studies, the extent of worker and public exposure to styrene emissions remains inadequately known. In 2023, another study conducted by Matthews et al. [9] showed that most of the coatings commonly used in practice for water and steam cure installations allow styrene to break through in a week or less. Beigvand et al. [10] reported that peak styrene concentrations during CIPP installations were observed at the liner truck openings and directly above the termination maintenance hole during the curing process.

Ra et al. [11] highlighted growing concerns regarding styrene emissions from CIPP installations due to their potential health and environmental risks. The resin curing process, which involves elevated temperatures and chemical reactions, often leads to the release of volatile organic compounds (VOCs), mainly styrene, into the surrounding environment. More than one hundred air contamination incidents have been linked to CIPP manufacturing sites, with limited information available on the chemicals emitted and their environmental and health impacts. According to the California Department of Public Health [12], over 130 exposure incidents related to CIPP installations have been reported across thirty states. These incidents highlight concerns regarding the migration of styrene and other hazardous vapors into nearby buildings through openings such as windows and doors, posing potential health risks to occupants; the extent of vapor migration is highly variable and influenced by subsurface conditions, project size, and structural characteristics of adjacent buildings [13].

Ajdari [14] documented VOC emissions during three CIPP sewer pipe rehabilitation projects. Measurements taken approximately 25.4 cm inside the maintenance hole (MH) revealed styrene levels exceeding occupational exposure limits during curing, with no other VOCs detected at measurable quantities using the equipment available. Sendesi et al. [15] also conducted a comprehensive investigation on VOC emissions at CIPP installation sites, highlighting the influence of factors such as location, wind direction, and worksite activity on emission levels. The findings revealed high concentrations of styrene in the air, which often concealed the detection of other VOCs. Subsequent research by Sendesi et al. [16] estimated that a single CIPP process could emit tens of tons of VOCs, from the initial resin composite preparation through the curing process. These emissions pose challenges to worker safety and the health of nearby populations, underscoring the need for robust monitoring and mitigation strategies.

A comprehensive review of CIPP emissions by Najafi et al. [17] recognized significant methodological deficiencies in earlier studies, emphasizing the need for standardized approaches to accurately evaluate and control VOC releases. Recent research on steam-cured cured-in-place pipe (CIPP) installations has highlighted several important findings regarding styrene emissions, including the following: (1) styrene is frequently detected at CIPP sites and is considered a compound of concern due to its associated health risks. (2) Real-time air monitoring using photoionization detectors (PIDs) has proven essential for supporting on-site decision-making and enhancing worker safety through immediate exposure assessment. (3) Although workers’ 8 h time-weighted average (TWA) exposures to styrene generally remain within the permissible limits established by occupational health agencies, recent findings indicate that short-term (lasting less than 15 min), task-specific exposures may exceed the Short-Term Exposure Limit (STEL) of 100 ppm; therefore, to ensure adequate protection during high-emission phases of the installation process, further investigation is required [18].

Although various VOCs, including acetone, benzene, styrene, 2-butanone, ethylbenzene, propene, and o-xylene, have been detected during the CIPP installation process, styrene consistently appears at concentrations exceeding regulatory thresholds. This study was conducted to improve the understanding of styrene emissions associated with cured-in-place pipe (CIPP) sewer rehabilitation projects. The specific objectives of the research were to (1) quantify styrene concentrations emitted during CIPP installations using different resin types and curing methods; (2) assess potential occupational health impact by comparing the measured styrene concentrations to established exposure limits established by Occupational Safety and Health Administration (OSHA), National Institute for Occupational Safety and Health (NIOSH), and the United States Environmental Protection Agency USEPA; (3) evaluate the influence of key operational parameters—including curing method, temperature, duration, pipe dimensions, and environmental conditions—on styrene emission levels; and (4) examine the dispersion of styrene concentrations to determine how emissions vary with distance from the source.

## 2. Materials and Methods

### 2.1. Site Selection

This study investigated six different cured-in-place pipe (CIPP) installations utilizing different resin liners and curing methods. The two primary resins used in these installations were polyester, which was saturated with styrene, and the other was non-styrene-based vinyl ester resin.

The first project (Site 1), conducted in June 2023, was in Garland, Texas. The host pipe was a 48.5-inch diameter clay pipe with a length of 1098 linear feet. This site was the longest and biggest pipe installation rather to others. The liner consisted of a felt tube saturated with polyester resin, with a thickness of 18 mm. The liner was delivered to the site in a refrigerated truck (called a liner truck in this paper) to maintain ideal conditions. During installation, the liner was inverted and inflated using water pressure, followed by curing with hot water. The second installation (Site 2) took place in October 2023 in Silver Spring, Maryland. The host pipe was an 8-inch clay pipe with a length of 155 linear feet. The liner material was a glass-reinforced tube saturated with polyester resin. In contrast with the other sites, this liner was kept in the wooden box, not a refrigerator. Installation involved pulling the liner into place and inflating it with air pressure, after which the curing process was completed using ultraviolet (UV) light. The third installation (Site 3) was completed in January 2024 in Forney, Texas. The host pipe, a clay pipe with a diameter of eight inches and a length of 672 linear feet, was rehabilitated using a felt tube liner saturated with polyester resin. The liner thickness was 6 mm. Installation involved the inversion of the liner with air pressure, followed by steam curing.

The fourth installation (Site 4) was completed in November 2024 in Flower Mound, Texas. The host pipe, a polyvinyl chloride (PVC) pipe with a diameter of twelve inches and a length of 464 linear feet, was rehabilitated using a standard tube liner saturated with vinyl ester resin. The liner thickness was 6 mm. The liner was inverted and inflated using water pressure, followed by curing with hot water. The fifth (Site 5) installation was in Washington, DC. It was completed in January 2024. The host pipe was an 18-inch-diameter vitrified clay pipe. The length of the installation was fifty-four feet, but the terminal discharge maintenance hole (MH) was in the valley, and the installation line had a very sharp slope. A 13 mm-thick liner saturated with vinyl ester resin (styrene-free) was used in this installation. Installation involved the inversion of the liner with water pressure, followed by hot water curing.

The sixth installation (Site 6) was in Washington, DC. It was completed in May 2024. The host pipe was a 10-inch diameter vitrified clay pipe. The length of the installation was 350 feet. A 4 mm-thick liner saturated with vinyl ester-Styrene free resin was used in this installation. Installation involved pulling the liner into place and inflating it with air pressure, after which the curing process was completed using ultraviolet (UV) light. Table 1 provides a comprehensive summary of the characteristics of these six CIPP installations, detailing the liner properties, host pipe specifications, and curing methods.

### 2.2. Resin Ingredient

In a study conducted by Li et al. [19] on the investigation of chemical emissions into both air and water from ultraviolet (UV)-cured CIPP installations, significant quantities of volatile compounds were detected in uncured resin tubes, ranging from 2.8% to 13.2% by weight. In contrast, cured CIPP samples exposed to elevated temperatures (120 °C and 160 °C) retained between 1.0% and 6.8% by weight of volatile constituents.

This study further categorized resin compositions. Sites 1 and 3 employed polyester-styrene-based resins, containing less than 45% styrene, less than 30% hydrous magnesium silicate (talc), less than 18% organic peroxide, less than 1.5% butyl cyclohexanol, and trace amounts (less than 1%) of quartz, alkanes, isododecane, and titanium dioxide by weight. The vinyl ester non-styrene-based resins utilized at Sites 5 and 6 were formulated with 25–65% vinyl ester polymer, 10–35% unsaturated polyester polymer, 15–35% hydroxyethyl methacrylate, 5–15% talc, and 1–10% dipropylene glycol diacrylate. At Site 4, a vinyl ester resin containing less than 45% styrene, up to 25% various catalysts, and less than 0.5% titanium dioxide was used. Data on resin composition for Site 2 is not available.

### 2.3. Data Collection

To monitor CIPP emissions during this study, two approaches were employed: (1) real-time monitoring utilizing Photoionization Detectors (PIDs) for continuous, on-site measurement of VOCs concentrations; and (2) laboratory analysis employing air sampling via Summa canisters to capture ambient air for laboratory-based determination of time-weighted average (TWA) styrene concentrations and workers’ sorbent tube samples.

#### 2.3.1. Real-Time Monitoring

Real-time air monitoring in this study was conducted using Honeywell MiniRAE 3000 Photoionization Detectors (PIDs), capable of measuring volatile organic compounds (VOCs) within a range of 0–99.9 ppm at a resolution of 0.1 ppm, utilizing a 10.6 eV lamp. Calibration was performed using isobutylene (IBE) gas as a surrogate standard, and styrene concentrations were calculated by applying a styrene-specific conversion factor. Three PIDs were strategically deployed: two for continuous monitoring, positioned upwind and downwind of the insertion maintenance hole (MH) near the liner truck, with measurements averaged every 15 min; and a handheld PID for periodically monitoring (every 30 min) at key locations, including 4 inches above the terminal discharge maintenance hole (MH), approximately five feet downwind of the work zone, and at lateral connections or passthrough maintenance holes if present. All PIDs were equipped with water trap filters to minimize the effects of high moisture levels anticipated during the curing process. Monitoring was conducted across the baseline (before installation) and during lining and curing phases of the CIPP process.

#### 2.3.2. Laboratory Analysis

To determine the time-weighted average (TWA) concentrations of styrene, air sampling was conducted using Summa canisters. The samples were analyzed in the laboratory using the EPA TO-15 method, which employs gas chromatography/mass spectrometry (GC/MS) for compound identification and quantification. The method specifies performance criteria rather than fixed detection limits, with laboratories required to establish compound-specific method detection limits (MDLs) through replicating low-level analyses. In this study, the reported MDL for styrene was 0.7 ppb. The method typically achieves accuracy within ±30% of the true concentration and precision of ≤25% relative standard deviation, providing reliable quantification of VOCs in both ambient and indoor air. Summa canisters were purposely installed to measure VOC concentrations within and around the cured-in-place pipe work area at each site. Sampling was performed during three key phases: baseline monitoring, liner installation, and curing at multiple locations, including upwind and downwind of the insertion maintenance hole and above the termination maintenance hole. To evaluate the influence of proximity to exhaust points on styrene concentrations, an additional canister was positioned within five feet downwind of the exhaust point in the active work zone.

Furthermore, worker sampling was conducted for two workers at each site over an 8 h work shift. The selected workers participated in various critical activities, including opening the liner truck to unload the uncured liner, feeding the liner into the inlet maintenance hole, inflating the liner, and monitoring the curing process at both the inlet and terminal maintenance holes. These tasks ensured that the sampling covered a broad range of potential exposure scenarios during the CIPP process. Passive worker samples are versatile for sampling from 15 min for short-term exposure limit (STEL) sampling and 8 h for TWA sampling to 14 days, depending on the sampling environment. The charcoal sorbent bed of each passive sampler was analyzed in the laboratory following a modified EPA TO-17 method. The VOCs were chemically extracted using carbon disulfide, and an aliquot of the extract was injected into a GC/MS for identification and quantification of VOCs.

#### 2.3.3. Weather Conditions

Climate plays a significant role in the dispersion of air emissions; therefore, it was important to try to capture sites with a range of climates so that study results could be compared across sites. At each site, wind speed, wind direction, temperature, and relative humidity were continuously measured and recorded. These measurements were taken throughout the entire period, during which concentration measurements were conducted. This data provided crucial context for understanding the dispersion and concentration of VOCs and other chemicals during the CIPP process. Table 2 summarizes weather condition data at six CIPP site installations.

## 3. Styrene Exposure Limits

The primary sources of styrene (CAS No. 100-42-5) in the ambient air are industrial activities and motor vehicle exhaust, with typical airborne concentrations around one part per billion (ppb). However, for individuals who smoke, cigarettes can be a main source of styrene exposure [20]. Due to its rapid biodegradation and volatility, styrene levels in surface water and groundwater are extremely low, often less than 1 microgram per liter (μg/L) or even undetectable [21]. This suggests that the environmental presence and persistence of styrene in aquatic systems is limited compared to its potential for airborne exposure.

During the CIPP installation process, the addition of styrene helps to reduce the viscosity of the resin, making it easier to manage and apply within the pipe. Importantly, styrene also plays a vital role in enabling the resin to cure from a liquid to a solid state through a process known as “cross-linking,” where the molecular chains of the polyester are bonded together [4].

The exposure limits for styrene are established and published by three prominent organizations: the United States Environmental Protection Agency (EPA), the Occupational Safety and Health Administration (OSHA), and the National Institute for Occupational Safety and Health (NIOSH). These exposure limit guidelines are designed to address a variety of real-world exposure scenarios and protect workers and the public from the potential health effects of styrene, which can range from transient discomfort to life-threatening consequences depending on the concentration and duration of exposure.

The Acute Exposure Guideline Levels (AEGLs), published by the EPA, establish threshold exposure limits for hazardous substances, including styrene, to protect the general population, including susceptible individuals. These guidelines are categorized into three levels based on the severity of potential health effects. AEGL-1 represents the airborne concentration above which individuals may experience notable discomfort, irritation, or mild, non-sensory effects. However, these effects are transient, reversible, and not disabling upon the end of exposure. AEGL-2 signifies the airborne concentration above which the general population, including sensitive individuals, could experience irreversible or other serious, long-lasting adverse health effects, or impaired ability to escape hazardous environments. AEGL-3 defines the airborne concentration above which the general population could face life-threatening health effects or death. These thresholds provide a framework to evaluate and manage public exposure to styrene under various scenarios. Specific AEGL values for styrene, as determined by the EPA, are detailed in Table 3 [22].

OSHA, the federal agency responsible for workplace safety, has established Permissible Exposure Limits (PELs) for styrene. The OSHA PEL for styrene is defined as an 8 h time-weighted average (TWA) concentration of 100 parts per million (ppm). Additionally, OSHA specifies an acceptable ceiling concentration of 200 ppm and a 5 min maximum peak concentration of 600 ppm, which should never be exceeded within any 3 h work period [23].

The NIOSH has also established exposure limits for styrene, focusing on short-term and long-term occupational exposure scenarios. The NIOSH Recommended Exposure Limit (REL) includes a Short-Term Exposure Limit (STEL) of 100 ppm for exposures typically lasting less than 15 min. The REL TWA, reflecting the Recommended Exposure Limit of Time-Weighted Average concentration for up to a 10 h workday over a 40 h workweek, is set at 50 ppm. Furthermore, NIOSH defines an Immediately Dangerous to Life or Health (IDLH) concentration of 700 ppm, which represents a threshold where individuals may experience severe disorientation, respiratory distress, or loss of ability to escape, emphasizing the critical need for strict exposure controls [24].

### Health Effects of Exposure to Styrene

While the USEPA has not yet classified styrene as a carcinogen, several studies have suggested a potential association between styrene exposure and the development of leukemia and lymphoma. The EPA is currently conducting an Integrated Risk Information System (IRIS) review to make a final determination regarding styrene’s carcinogen classification. In their review of the available scientific data on the carcinogenicity of styrene, an expert panel concluded that the evidence for styrene’s carcinogenicity in humans is only “suggestive.” Although epidemiological studies have not provided clear evidence of increased cancer risk among workers exposed to styrene, animal studies have demonstrated an increased occurrence of lung tumors in mice exposed to the chemical. While styrene does not appear to be directly genotoxic, it is metabolized in both the lung and liver to styrene oxide, a genotoxic agent. As a result, the panel determined that styrene’s carcinogenic potential in humans cannot be definitively ruled out [19]. However, the research conducted by Cruzan et al. [25] shows that the mouse lung tumors identified in previous studies may not be relevant to assessing the human cancer risk associated with styrene exposure.

Banton et al. [26] noted that styrene exposure may interact with noise exposure, underscoring the importance of implementing noise reduction measures in occupational settings where styrene is used. Styrene and other simple aromatic chemicals, such as toluene, ethylbenzene, and xylenes, have been linked to hearing loss in the workplace, particularly in conjunction with noise exposure.

In 2018, an OSHA investigation revealed that a 22-year-old man died on an Illinois worksite partly due to chemical overexposure. Drowning with styrene toxicity was listed as a significant contributing factor [27].

In a study conducted by Bertke et al. [28], occupational exposure to styrene among workers was found to be associated with an elevated risk of lymphohematopoietic cancers (LHC), whereas no significant association was observed between styrene exposure and the incidence of lung cancer. According to the National Center for Biotechnology Information [29], long-term exposure to styrene can cause skin problems such as blistering and dermatitis, due to its drying effect. Studies have also reported liver issues, including increased bile acids and enzyme activity, as well as reproductive problems like lower birth rates and increased frequency of spontaneous abortions in female workers. Additionally, workers exposed to styrene have shown higher rates of certain cancers, particularly leukemia and lymphoma, with possible links to pancreatic and esophageal cancer. A study by Ska et al. [30] on the acute neurotoxic effects of short-term peak exposures to styrene showed that short-term exposures to styrene did not produce significant neurotoxic effects under the controlled conditions.

## 4. Results and Discussion

**Figure 1:**
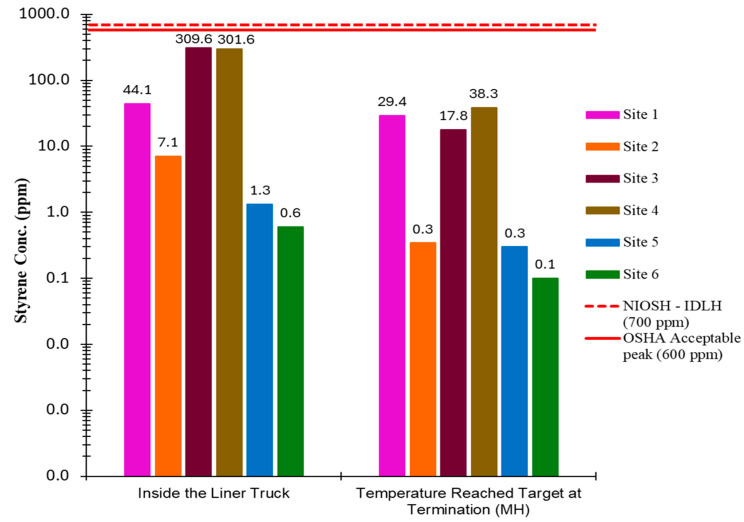
Maximum styrene instantaneous concentration.

Figure 1 illustrates the maximum instantaneous concentration of styrene, measured by PID, at six CIPP sites inside the liner truck (opening truck) and during the curing process above the terminal maintenance hole. PID isobutylene VOC readings were converted to styrene concentrations by applying a correction factor of 0.43, as recommended in Honeywell Technical Note TN-106 [31] for PIDs equipped with a 10.6 eV lamp. This correction factor was used to estimate styrene concentrations in air emissions during the study.

The peak PID readings, represented in Figure 1, were recorded immediately upon opening the liner transportation truck, with maximum styrene concentrations of 309.6 ppm at site three and 301.6 ppm at site four. The maximum measurement at site 2, although the resin type is styrene-based, is 7.1 ppm. During the curing process, the highest instantaneous styrene concentrations were observed at Sites 1 and 4, measuring 29.4 ppm and 38.3 ppm, respectively. All recorded instantaneous styrene concentrations were below the NIOSH Immediately Dangerous to Life or Health (IDLH) threshold of 700 ppm and the Occupational Safety and Health Administration (OSHA) permissible maximum peak concentration of 600 ppm. The observed peak concentrations were short-term, with durations not exceeding one minute.

**Figure 2:**
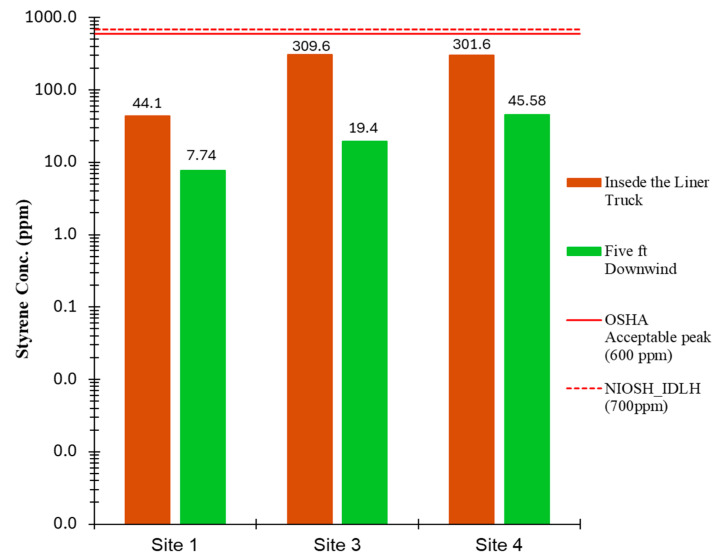
Instantaneous styrene concentrations were measured upon opening the liner truck.

To investigate the styrene concentration in the work zone perimeter and its possible impact on workers, these measurements were taken downwind of the liner truck. Figure 2 presents a comparison of styrene concentrations measured inside the liner truck and at a location five feet downwind for three sites with elevated results. At Sites 1 and 3, downwind concentrations decreased significantly to 7.74 ppm and 19.4 ppm, respectively, while at Site 4, the concentration reached 45.58 ppm. It is important to note that this value represented an instantaneous one-minute measurement and nevertheless remained below the established exposure limits.

**Figure 3:**
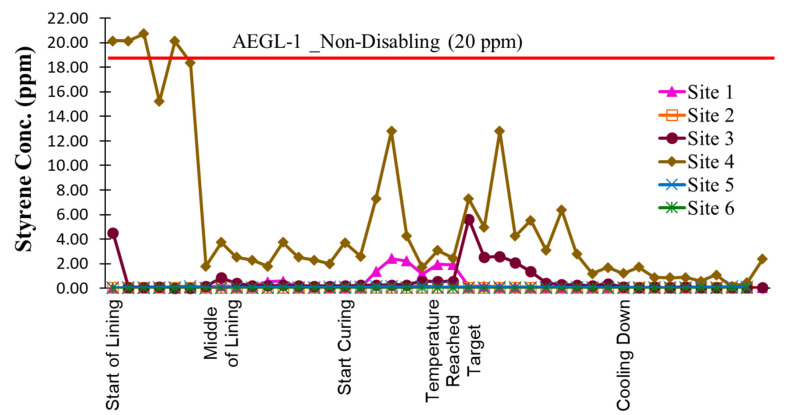
Styrene 15 min average concentration at the downwind of insertion MH.

Figure 3 describes the 15 min average continuous measurement of styrene in parts per million (ppm) at the downwind side of the insertion maintenance hole during the CIPP installation. The figure also highlights the AEGL-1 (10–30 min) exposure limit of 20 ppm. As shown in this figure, the measurements at sites 1,2,3,5, and 6 are below the acute exposure guideline level 1, while the styrene concentration at the start of the lining process, when the truck was open at site 4, exceeds the AEGL-1, which causes notable discomfort, irritation, or mild, non-sensory effects for workers who work near the liner truck. All six sites follow the same trend and have the highest result during the curing process, and after the temperature reaches the target cooking point, the average styrene concentrations at this point are 2.44 ppm, 0.04 ppm, 5.64 ppm, 12.8 ppm, 0.15 ppm, and 0.0027 ppm for sites one through six, respectively. At sites 2,5, and 6, the PID readings remained consistently below 0.2 ppm throughout the entire process, reflecting the absence of significant volatile organic compound (VOC) releases from the UV curing method and non-styrene-based resin liner.

**Figure 4:**
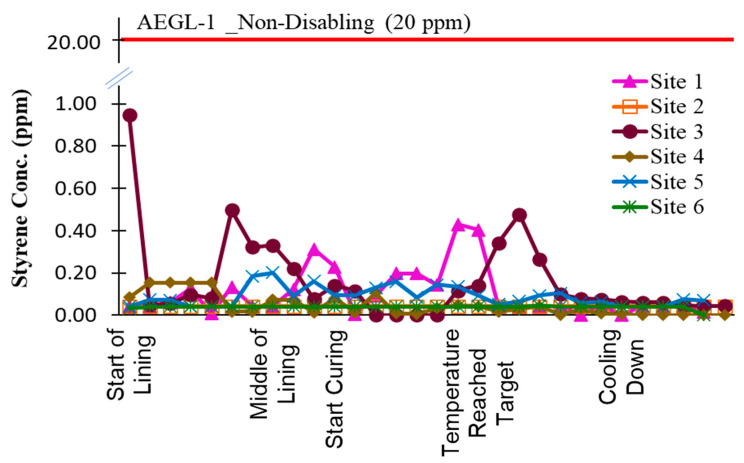
Styrene 15 min average concentration at the upwind of insertion MH.

Figure 4 presents 15 min average continuous styrene measurements taken at the upwind side of the insertion maintenance hole for all six sites. Across all installations, the upwind styrene concentrations were consistently below 0.5 ppm. At Site 3, the peak reading of 0.95 ppm corresponded with the start of the lining process.

To assess the time-weighted average concentration of styrene, two sorbent tubes were attached to workers who worked in the work zone perimeter from the start of work to the end of installation. The reported results represent average concentrations over the time-weighted period (TWA) and sampling durations specified in Table 4. Sampling durations at most sites ranged from 4 to 10 h, depending on the total liner length and the duration of installation and curing at each site. At Site 1, which had the longest shot lengths and the largest pipe diameter, the installation process extended to approximately 26 h. Two different workers carried the sorbent tubes, with a shift change occurring after 13 h, at which point the samples were transferred to the incoming workers. Sample #2 at Site 1 recorded the highest average styrene concentration of 13.85 ppm; this sample was held by workers positioned near the termination maintenance hole throughout the curing process. However, because the sample was not held continuously by a single worker and included a shift change, it may not serve as a fully representative measure of individual worker exposure. The installation at Site 1 comprised 10 h for liner inversion and inflation, followed by 16 h for curing. The curing phase included 8 h of boiler warm-up to reach the target temperature of 160 °F and 8 h of active curing at that temperature. Worker Sample #2 at Site 3 recorded a styrene concentration of 8.22 ppm. Notably, Site 3 utilized steam curing and had the shortest installation duration among all sites, approximately 4 h in total, including 1.5 h for the lining process, 1.5 h for the curing phase, and 1 h for cooling. All other worker samples recorded styrene concentrations below 3 ppm. Across all sites, none of the time-weighted average styrene concentration recorded with worker samples exceeded applicable occupational exposure limits, including NIOSH, OSHA, and EPA.

**Figure 5:**
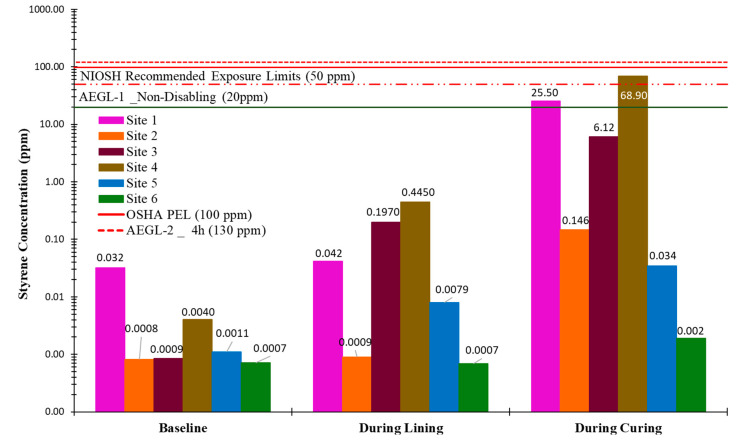
Time-weighted average styrene concentration above termination MH.

Figure 5 illustrates the canister sampling results for styrene concentrations directly above the termination maintenance hole. The results indicate that styrene concentrations were higher during the curing phase compared to the lining phase for all six sites. Site four recorded the highest styrene concentration of 68.9 ppm during the curing process. This concentration exceeded both the AEGL-1 threshold of 20 ppm and the NIOSH REL of 50 ppm. According to the Centers for Disease Control and Prevention (CDC), exposure at or above these levels may irritate the eyes, nose, and respiratory system; headache; lassitude (weakness, exhaustion); dizziness; confusion; malaise (vague feeling of discomfort); drowsiness; unsteady gait; narcosis; defatting dermatitis; possible liver injury and reproductive effects. At Site 1, the styrene concentration measured above the termination maintenance hole during the curing process was 25.5 ppm. This value exceeds the AEGL-1 but remains well below other relevant exposure limits, including the NIOSH REL, the OSHA PEL, and the AEGL-2 (8 h) threshold of 130 ppm. At Site 3, despite the use of steam curing and a relatively high emission rate, the styrene concentration measured during curing was 6.12 ppm, significantly below all applicable exposure limits. For all other sites, styrene concentrations during the curing phase were measured below 1 ppm.

**Figure 6:**
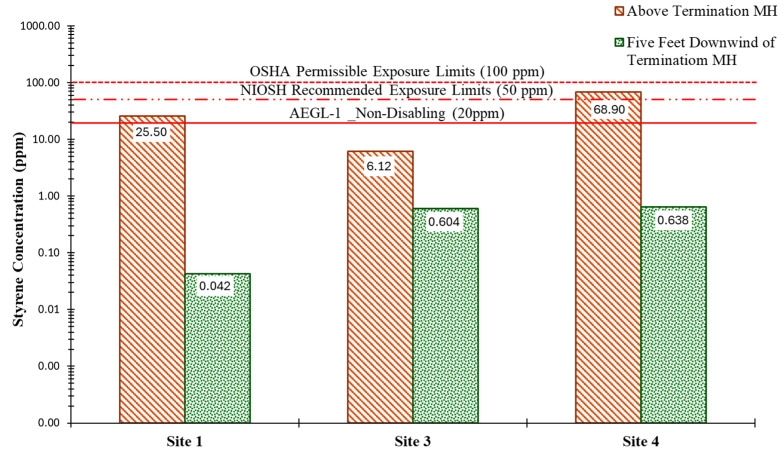
Time-weighted average styrene concentration during the curing process.

Figure 6 compares styrene concentrations measured directly above the termination maintenance hole, along with those at five feet downwind, as it evaluates styrene dispersion within the work zone, at the three sites where measurements were observed to peak. As presented, styrene concentrations decreased noticeably at each downwind location. This indicated effective dispersion of styrene emissions from the emission source. Field observations showed that the pollutant concentrations dissipated entirely at 20 feet from the termination maintenance hole, where the PID readings dropped to zero.

**Figure 7:**
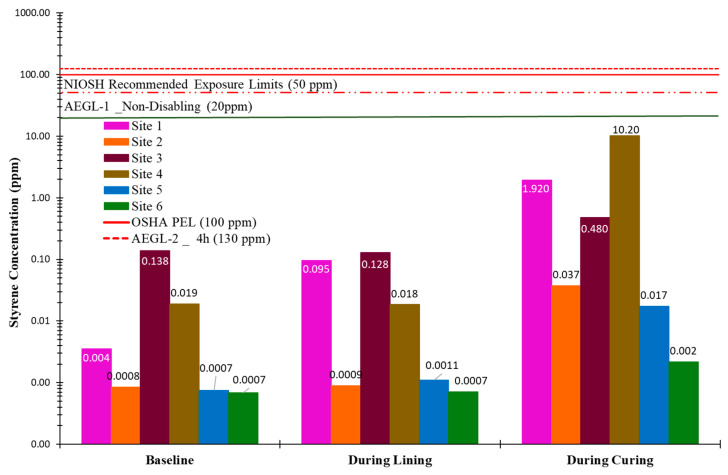
Time-weighted average styrene concentration—upwind of insertion MH.

**Figure 8:**
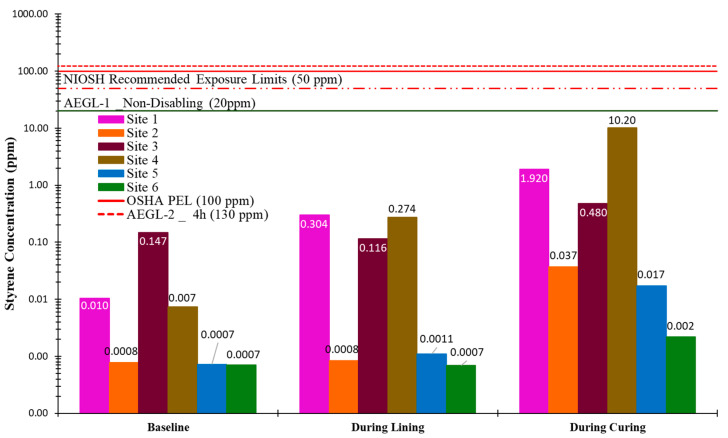
Time-weighted average styrene concentration—downwind of insertion MH.

Figure 7 and Figure 8 present the canister sampling results for styrene concentrations at the upwind and downwind locations of the insertion maintenance hole, respectively. Baseline samples for all sites were collected over a continuous 1 h period. However, the sampling durations for the lining and curing phases varied depending on the project specifications and curing methods. As illustrated, the time-weighted average (TWA) styrene concentrations at both the upwind and downwind locations of the insertion maintenance hole for all sites remained below established exposure limits. As expected, styrene concentrations downwind of the insertion point were higher than upwind, consistent with the direction of prevailing airflow and typical emission dispersion patterns.

## 5. Conclusions

This study was conducted to evaluate styrene emissions during CIPP installations and to investigate how resin type and site-specific variables influence VOC emissions. Instantaneous PID measurements indicated elevated styrene concentrations originating from uncured liners inside the liner truck. Field data collected from six CIPP projects across the U.S. revealed that uncured liners exhibiting initially significant styrene emissions produced elevated time-weighted average (TWA) values during the curing phase. However, one site demonstrated that even liners with minimal initial emissions could produce high TWA concentrations, highlighting the complexity of emission dynamics during curing.

EPA TO-15 analysis targeting specific tasks during installation revealed that elevated styrene concentrations can exist near the termination maintenance hole during the curing process. Notably, TWA styrene concentrations associated with styrene-based resin reached 25.5 ppm and 68.9 ppm at two sites, exceeding the AEGL-1 threshold (20 ppm) and the NIOSH REL (50 ppm), respectively. Additional samples indicated that styrene concentrations substantially decreased approximately five feet downwind and became undetectable at twenty feet. Worker exposure data obtained via sorbent tube analysis showed no exceedance of occupational exposure thresholds. While previous studies have identified steam curing as the worst-case scenario due to visible vapor plumes, the highest observed styrene concentrations in this study were associated with the use of a vinyl ester styrene-based resin cured with hot water. The absence of precise measurements of styrene content in each liner before installation limits the ability to attribute emission variations solely to the curing method. These findings indicate that emissions are influenced by the initial resin formulation, particularly styrene content. Accordingly, the use of low-styrene or styrene-free resins is recommended as an effective strategy to mitigate emissions.

In conclusion, while CIPP installations may pose minimal risk to the general public in open-air environments, they present potential health risks to workers in proximity to emission sources such as the liner truck and termination maintenance hole. Therefore, the use of appropriate personal protective equipment (PPE), adherence to established safety protocols for confined space entry, and education on the health hazards associated with CIPP liner ingredients are essential. Furthermore, to assess actual worker exposure, the implementation of biological monitoring is recommended, as it can also help evaluate the effectiveness of the protective systems and serve as a valuable approach for future verification of worker exposures. Given the complexity of emission dynamics during the curing process, this work is limited by the absence of verification for other VOCs that may be emitted or generated by curing reactions and additional chemical interactions during pipe repair. Additionally, considering the variability in environmental and worksite conditions, real-time air quality monitoring, and periodic assessment of worker exposures, particularly during tasks involving close contact with emission sources, are critical to ensuring a safe work environment.

## Figures and Tables

**Table 1:** Key characteristics of CIPP installations.

Environment	Site	Segment Length (LF)	Pipe Diameter	Liner Thickness (mm)	Curing Method	Resin Liner Type	Curing Duration (h)
			(in)				
Residential	1	1091	48.5	18	Hot-Water	Polyester (styrene-based)	9 h
Residential	2	155	8	3.5	UV Light	Polyester (styrene-based)	1.5 h
Residential	3	672	8	6	Steam	Polyester (styrene-based)	1.5 h
Residential	4	464	12	6	Hot-Water	Vinyl ester (styrene-based)	6 h
Residential	5	54	18	13	Hot-Water	Vinyl ester (styrene-free)	6 h
Residential	6	350	10	4	UV Light	Vinyl ester (styrene-free)	2.5 h

**Table 2:** Weather conditions.

Site	Wind Speed (mph)	Wind Direction	Min Temp (°F)	Max Temp (°F)	Humidity
					%
1	2.8	SW	68	92	70
2	3.5	NE	47.1	57.8	86.6
3	2.1	WNW-W	39	74	63
4	2.8	E-ESE	49	74	66.1
5	4	NE	42.2	56	91.1
6	5.5	SW-S	44.1	77	62

**Table 3:** Styrene EPA acute exposure guideline levels.

Guideline or Standard		Exposure Duration				
		10 min	30 min	1 h	4 h	8 h
		ppm	ppm	ppm	ppm	ppm
AEGL-1	Non-Disabling	20	20	20	20	20
AEGL-2	Disabling	230	160	130	130	130
AEGL-3	Lethal	1900	1900	1100	340	340

**Table 4:** Worker samples result.

Location	Sampling Time (h)	Report Limit (ppm)	Blank Sample (ppm)	TWA Styrene Concentration (ppm)			Exposure Limits (ppm)	
				Worker 1	Worker 2	Relative Percentage Difference (RPD)	NIOSH-REL TWA	OSHA-PEL TWA
Site 1	26	0.0003	ND	2.817	13.85	132.4%	50	100
Site 2	6	0.0012	ND	<0.0012	<0.0012	0%	50	100
Site 3	4	0.0026	ND	1.456	8.22	139.8%	50	100
Site 4	8	0.0008	ND	1.456	1.85	24.11%	50	100
Site 5	10	0.0006	ND	0.0026	0.0033	24%	50	100
Site 6	8	0.0014	ND	<0.0014	<0.0014	0%	50	100

## Data Availability

The raw data supporting the conclusions of this article will be made available by the authors on request.
